# BRM270 inhibits cancer stem cell maintenance via microRNA regulation in chemoresistant A549 lung adenocarcinoma cells

**DOI:** 10.1038/s41419-018-0277-7

**Published:** 2018-02-14

**Authors:** Taeho Kwon, Nisansala Chandimali, Do Luong Huynh, Jiao Jiao Zhang, Nameun Kim, Yesol Bak, Do-Young Yoon, Dae-Yeul Yu, Jae Cheol Lee, Meeta Gera, Mrinmoy Ghosh, Yang Ho Park, Dong Kee Jeong

**Affiliations:** 1 Laboratory of Animal Genetic Engineering and Stem Cell Biology, Subtropical/Tropical Organism Gene Bank, Jeju National University, Jeju, Republic of Korea; 2 Laboratory of Animal Genetic Engineering and Stem Cell Biology, Department of Animal Biotechnology, Faculty of Biotechnology, Jeju National University, Jeju, Republic of Korea; 3 Department of Bioscience and Biotechnology, Bio/Molecular Informatics Center, Konkuk University, Seoul, Republic of Korea; 4 Disease Model Research Laboratory, Genome Editing Research Center, Korea Research Institute of Bioscience and Biotechnology (KRIBB), Daejeon, Republic of Korea; 5 Asan Institute for Life Sciences, Asan Medical Center, College of Medicine, University of Ulsan, Seoul, Republic of Korea; 6 BRM Institute, Seoul, Republic of Korea

## Abstract

Chemotherapy is a standard treatment for non-small-cell lung cancer (NSCLC). However, the dose-limiting toxicity of drugs and the development of chemoresistance are major clinical challenges to successful management of NSCLC. Asian traditional medicine is gaining global attention as a non-toxic alternative to chemotherapy. BRM270 is an extract formulated from seven Asian medicinal plants that has been shown to inhibit tumor cell proliferation in diverse cancer types. We previously demonstrated that BRM270 suppresses tumorigenesis by negatively regulating nuclear factor-κB signaling in multidrug-resistant cancer stem cells (CSCs). In this study we report that the growth, migration, and invasion of normal human lung adenocarcinoma cells and their chemoresistant derivatives was inhibited by BRM270 treatment. Notably, BRM270 was found to modulate CSC self-renewal and tumor-initiating capacity via positive regulation of the miRNA-128. Thus, combination therapy with miRNA-128 and BRM270 may be an effective treatment strategy for chemoresistant NSCLC.

## Introduction

Lung cancer is the most common type of malignancy and the leading cause of cancer-related mortality worldwide^1^. Non-small-cell lung cancer (NSCLC) accounts for approximately 85% of all cases and has a 5-year survival rate of only 15%^2^. Gefitinib and paclitaxel are reversible epidermal growth factor (EGF) receptor-specific tyrosine kinase inhibitors (EGFR-TKIs) and are considered as first-generation EGFR-TKIs; second-generation EGFR-TKIs are currently being developed to overcome drug resistance^3^, which is caused in part by cancer stem cells (CSCs). Gefitinib-resistant and paclitaxel-resistant cell lines derived from A549 human lung adenocarcinoma cells (A549/GR and A549/PTX, respectively) were recently established by repeated exposure to gefitinib^4, 5^, although their activity has not been investigated in the context of EGFR expression^6^. A549/GR and A549/PTX cells have greater sphere-forming capacity and higher expression of aldehyde dehydrogenase 1, cluster of differentiation (CD)133, C-X-C chemokine receptor (CXCR)4, and stemness-related factors such as Nanog, octamer-binding transcription factor (Oct)4, (sex determining region Y)-box (Sox)2, B cell-specific Moloney murine leukemia virus integration site (Bmi)-1, and Musashi-1 as compared to parent A549 cells^4, 5^. Nonetheless, there is a need for biomarkers that can be used to predict clinical outcome following chemotherapy and to identify patients at risk of developing drug resistance^7^.

Micro (mi) RNAs are small non-coding RNAs 19–22 nucleotides in length that bind to target mRNAs and target them for degradation^8^. miRNAs act as oncogenes or tumor suppressors to regulate tumor proliferation, invasion, apoptosis, and therapeutic resistance^7^. miR-128 is a typical tumor suppressor that is downregulated in many malignancies including lung cancer^9, 10^. The reduced level of miR-128 in NSCLC patients has been linked to tumor differentiation, pathological stage, and lymph node metastasis^9^. Recent evidence indicates that multidrug resistance arises as a result of accumulated genetic and epigenetic changes, and is mediated in part by miRNAs^7^. Additionally, miRNAs have been implicated in acquired gefitinib resistance in lung adenocarcinoma; miR‑128 can potentially reverse drug resistance^11^.

Asian traditional medicine has recently been gaining global attention owing to the low toxicity of medicinal herbs^12^. Various medicinal plant extracts have shown therapeutic efficacy against cancer—including angiogenesis and metastasis—without observable side effects^13^. We previously reported that BRM270, an extract formulated from seven medicinal plants, contains compounds that target the nuclear factor (NF)-κB signaling pathway^14^ and induce cell cycle arrest and apoptosis, as evidenced by the downregulation of the CSC marker CD133^14^.

Based on these observations, we investigated the effect of BRM270 in GR and PTX human NSCLC A549 cells and found that it suppressed tumorigenesis by directly targeting miR-128. Moreover, BRM270 was found to modulate epithelial–mesenchymal transition (EMT), CSC self-renewal, and the expression of stemness-related-genes in all cell lines examined. Our results indicate that BRM270 may be an effective treatment for chemoresistant NSCLC.

## Results

### BRM270 inhibits proliferation and induces apoptosis in A549 cell lines

**Fig. 1:**
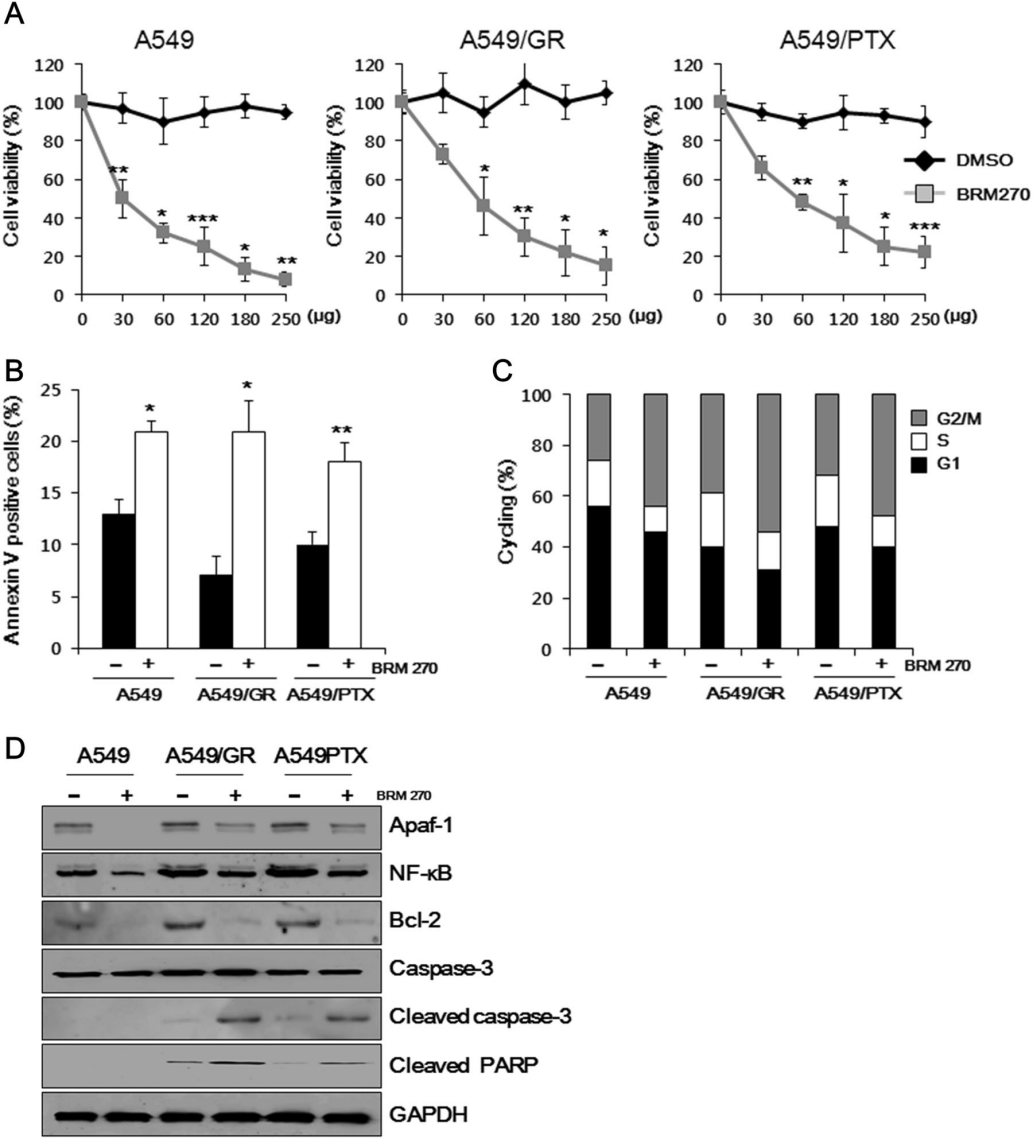
Effects of BRM270 on cell viability in A549 cell lines. **a** Approximately 2 × 10^4^ cells/well were seeded in 96-well plates and treated with BRM270 (0, 30, 60, 120, 180, and 250 µg/ml) for 48 h; 0.1% dimethylsulfoxide in medium was used as a control. **b** Percentages of Annexin V-positive cells. **c** Cell cycle distribution. **d** Expression of apoptosis-related factors. Data represent mean ± SEM (*n* = 5 per group). *P < 0.05, **P < 0.01, ***P < 0.001

We previously showed that BRM270 is cytotoxic against multidrug-resistant CSCs in vitro and that this effect was exerted by inhibiting the growth of OCT3/4 and CD133^+^ populations^14^. Here we investigated whether BRM270 has similar effects in A549 cells, including the A549/GR and A549/PTX chemoresistant cell lines. The anti-proliferative effects of BRM270 were evaluated with the 3-(4,5-dimethylthiazol-2-yl)-2,5-diphenyltetrazolium bromide assay. Exposure to various concentrations of BRM270 (0, 30, 60, 120, 180, and 250 µg/ml) for 24 h decreased cell viability in a dose-dependent manner in all cell lines examined (Fig. 1a). Additionally, to gain further insights on the effect of BRM270 compared to existing chemotherapy, we compared the effects of BRM270 with Gemcitabine, an existing chemotherapy that is used to treat many types of cancers, including non-small-cell lung cancers. A MTT assay revealed that Gemcitabine decreases the viability of A549, A549/GR, and A549/PTX cells in a dose-dependent manner after 24 h of treatment with different Gemcitabine concentrations (0.0001, 0.001, 0.01, 0.11, and 10 μM). Another MTT assay revealed that the viability of BRM270-treated A549/PTX cells is greatly downregulated than Gemcitabine-treated A549/PTX cells. Gemcitabine-treated A549/GR cells showed higher viability reduction compared to BRM270-treated A549/GR cells. Side effects did not result in both BRM270 and Gemcitabine as we checked on human bone marrow cells (hBMCs) (Supplementary Figure 1a and b). Moreover, the percentage of Annexin V-positive cells was induced by BRM270 (120 µg/ml) treatment as compared to untreated control cells (Fig. 1b). BRM270 treatment also reduced the G1 phase fraction and cause G2/M arrest (Fig. 1c). We examined the expression of pro-apoptotic (Apoptotic protease-activating factor [Apaf]-1) and anti-apoptotic (p65, phosphorylated NF-κB, and B cell lymphoma [Bcl]-2) factors, as well as the downstream effector cleaved caspase-3 by western blotting. BRM270 induced the cleavage of caspase-3 and PARP in A549, A549/GR, and A549/PTX cells (Fig. 1d). These data suggest that BRM270 treatment induces apoptosis and G2/M arrest in normal and chemoresistant A549 cells by inhibiting NF-κB/Bcl-2 signaling.

### BRM270 suppresses the stem-like properties of CSC cells

**Fig. 2:**
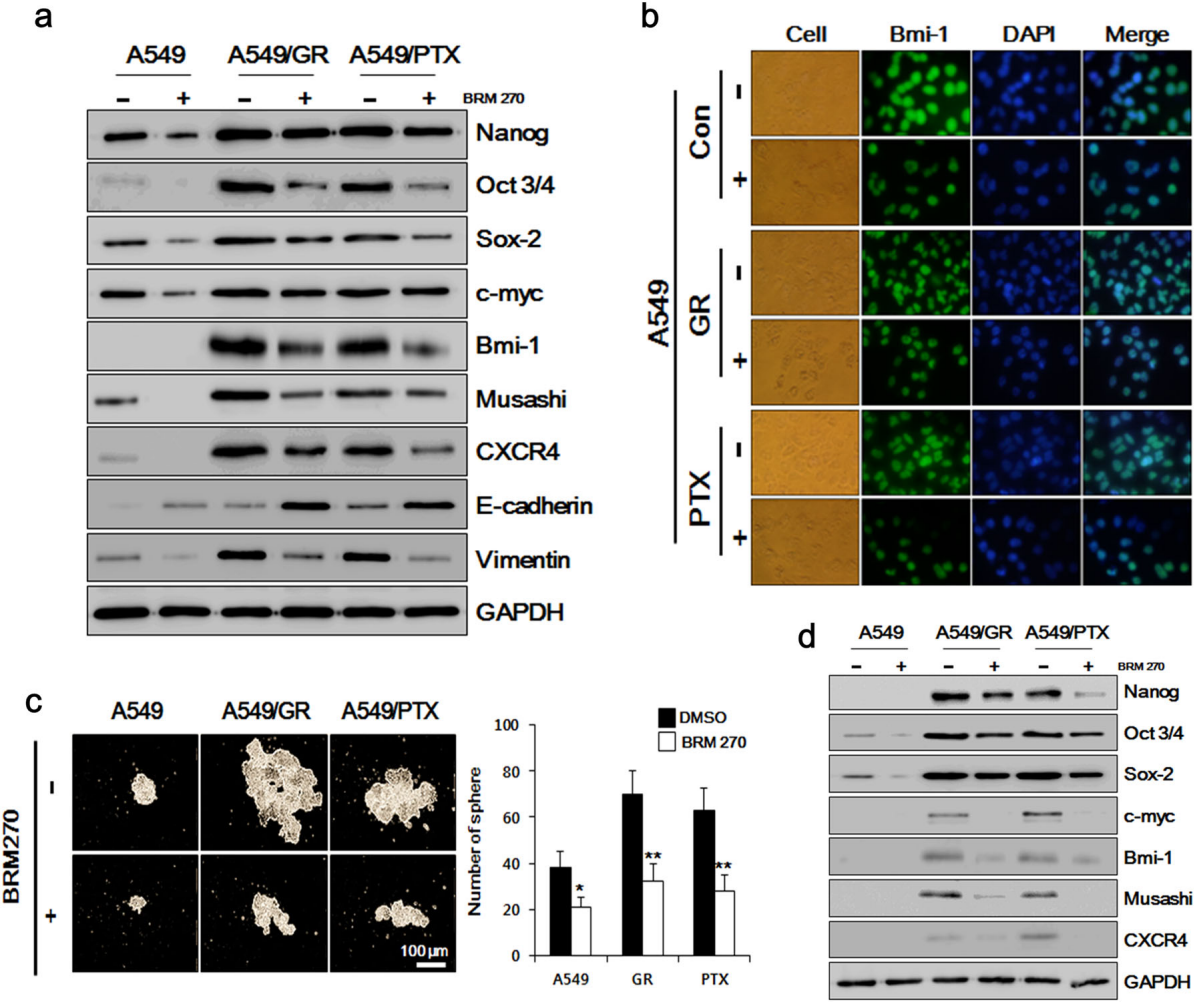
BRM270 impairs tumor sphere formation by A549 cells. **a** Expression levels of CSC markers and EMT signaling pathways in A549 cells treated with BRM270. **b** Representative images of A549 cells treated with BRM270 and labeled with an antibody against Bmi-1. **c** Number of spheres in A549 cells treated with BRM270. **d** Expression levels of CSC markers in A549 cell spheroids. Data represent the mean ± SEM (*n* = 5 per group). *P < 0.05, **P < 0.01

GR and PTX A549 cells exhibit stem-like features^4–6^. We investigated whether BRM270 treatment affects these characteristics in normal and chemoresistant A549 cells. The stemness factors Nanog, Oct 3/4, Sox-2, c-myc, Bmi-1, Musashi-1, and CXCR4 were downregulated in the presence of BRM270 relative to untreated cells, as determined by western blotting (Fig. 2a) and immunocytochemistry (Fig. 2b). BRM270 also induced E-cadherin while suppressing Vimentin expression in all three cell lines (Fig. 2a). These data indicate that BRM270 can inhibit EMT and thus metastatic progression.

A549/GR and A549/PTX cells exhibited increased sphere-forming capacity after 14 days of culture as compared to A549 cells. However, sphere size and number were reduced by BRM270 treatment relative to untreated cells (Fig. 2c). A western blot analysis revealed that Nanog, Oct3/4, Sox2, c-myc, Bmi-1, Musashi-1, and CXCR4 were downregulated in the presence of BRM270 (Fig. 2d). Thus, BRM270 suppresses the stemness properties of A549 cells.

### BRM270 blocks cancer progression by inducing miR-128 expression

**Fig. 3:**
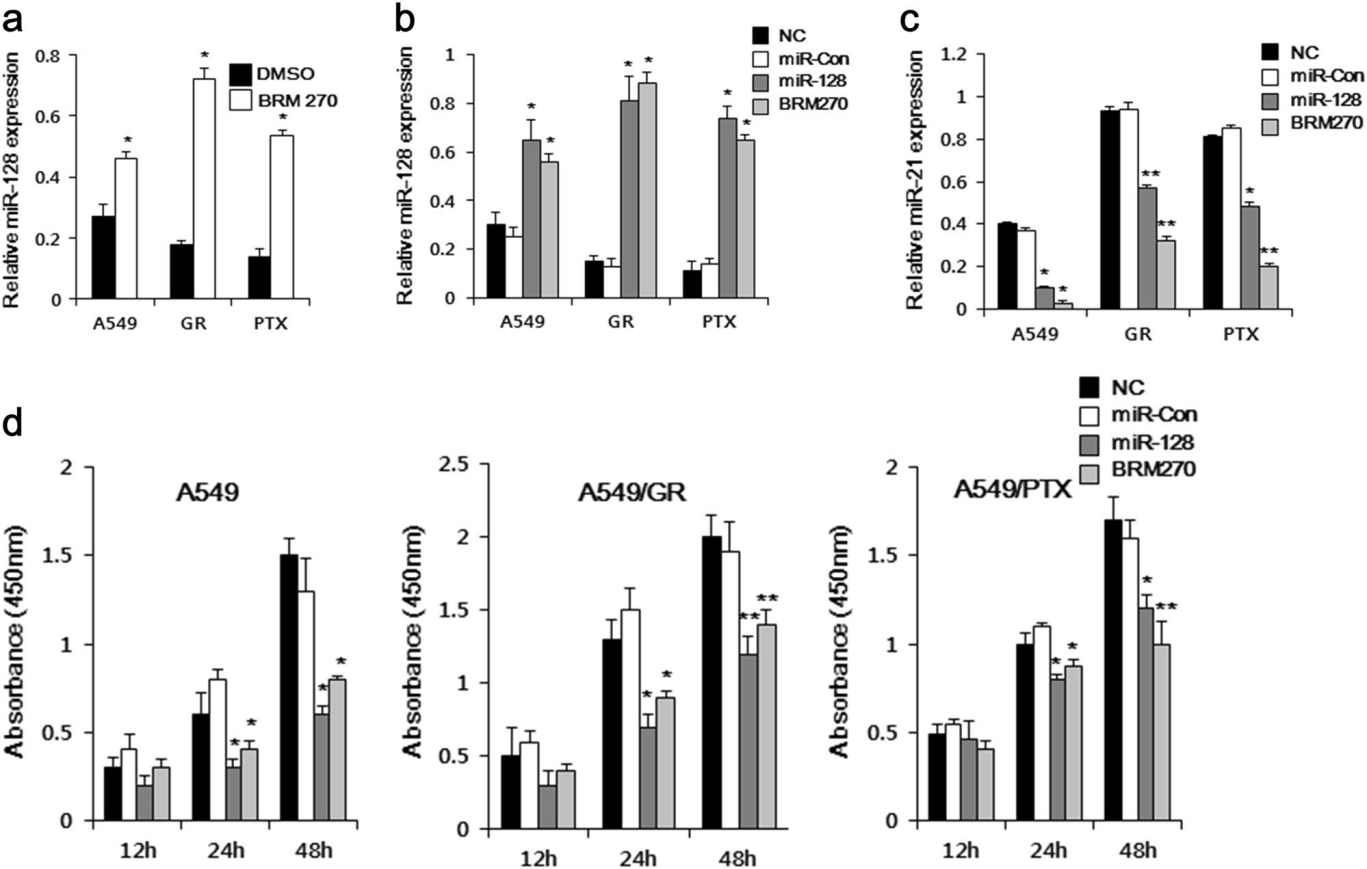
BRM270 induces miR-128 and inhibits miR-21 expression in A549 cell lines. **a** miR-128 level was increased in A549 cells treated with BRM270. **b** miR-128 and **c** miR-21 expression levels in A549 cells treated with miRNA128 and BRM270. **d** A549 cell proliferation at 12, 24, and 48 h after transfection and treatment with miRNA128 and BRM270, as determined with the 3-(4,5-dimethylthiazol-2-yl)-2,5-diphenyltetrazolium bromide assay. Data represent mean ± SEM (*n = *5 per group). *P < 0.05, **P < 0.01

It was previously reported that miR-128 was downregulated in lung tumor tissue, suggesting that miR-128 may play an important role in NSCLC progression and development^9^. MiR-128 modulates chemotherapeutic sensitivity in CSCs by targeting Bmi-1 and Musashi-1, and its reduced expression in cancer tissues is correlated with chemotherapeutic resistance and poor patient outcome^15, 16^. We observed that miR-128 expression was attenuated in the A549 cell line and its chemoresistant derivatives. However, BRM270 treatment increased miR-128 expression by 2–3 folds in A549, A549/GR, and A549/PTX cells (Fig. 3a, b). MiR-21 has been shown to modulate resistance to various chemotherapeutic agents including gefitinib in several cancer cell types^17^. Here we found that BRM270 blocked the activation of NF-κB, resulting in the inhibition of miR-21 expression (Fig. 3b), which was associated with reduced proliferation in A549 cell lines (Fig. 3d).

### BRM270 prevents the maintenance of the CSC phenotype via regulation of miR-128

**Fig. 4:**
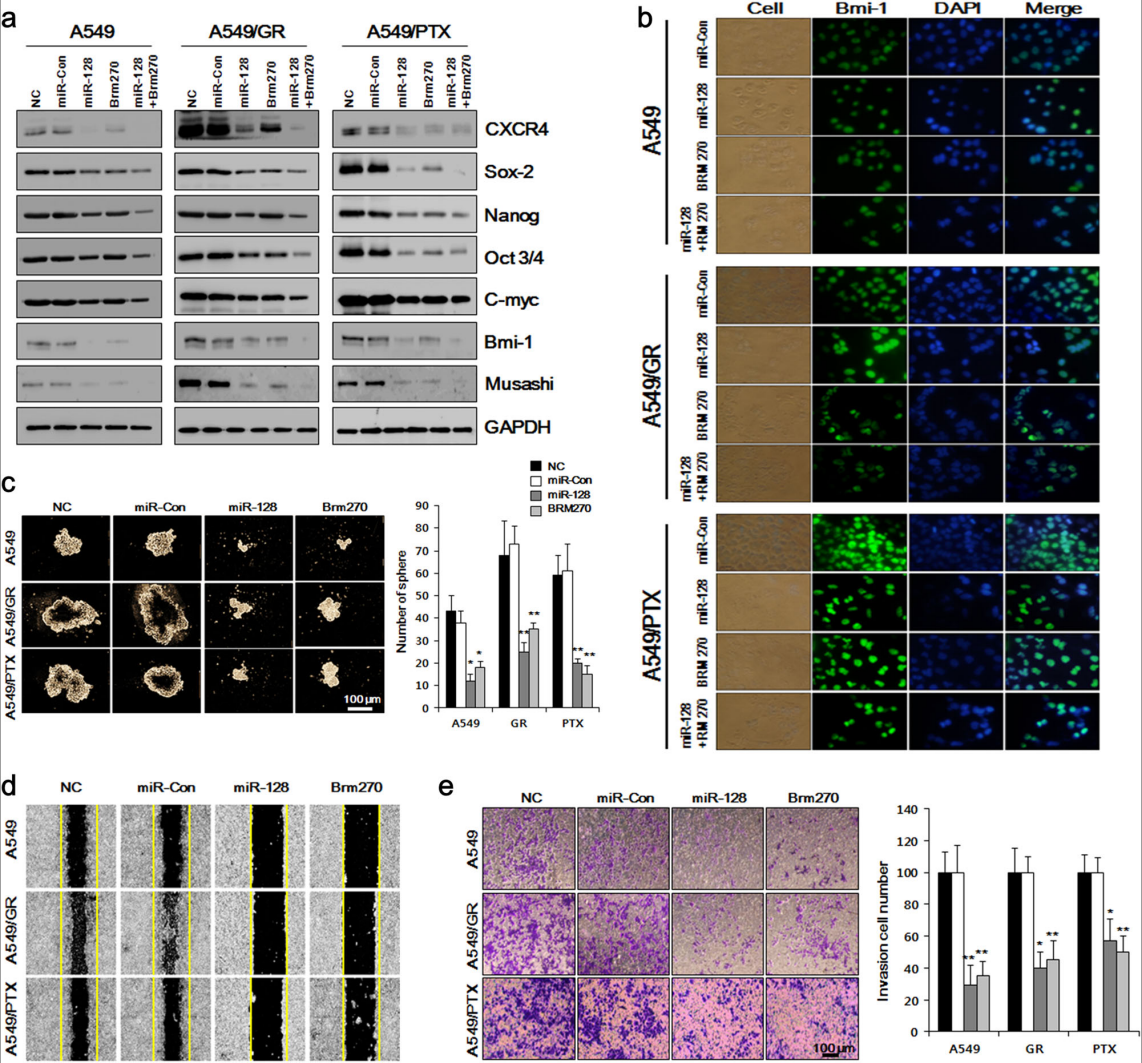
BRM270-induced miR-128 overexpression in A549 cell lines suppresses the expression of stemness genes and cell growth. **a** Expression levels of CSC markers in A549 cells treated with miR-128, BRM270, or both. **b** Representative images of A549 cells treated with miR-128, BRM270, or both and probed with an antibody against Bmi-1. **c** Number of A549 cell spheres following treatment with miR-128, BRM270, or both. **d** Wound healing and **e** invasion assays in A549 cells treated with miR-12, BRM270, or both. Data represent mean ± SEM (*n* = 5 per group). *P < 0.05, **P < 0.01

We investigated whether the combination of BRM270 and miR-128 could alter the protein levels of the stemness factors Nanog, Oct3/4, Sox2, c-myc, Bmi-1, Musashi-1, and CXCR4 in A549 cell lines. We found that BRM270+miR-128 more potently reduced the expression of all factors than either treatment alone, as determined by Western blotting (Fig. 4a) and immunocytochemistry (Fig. 4b). BRM270 also suppressed the formation of A549/GR and A549/PTX cell spheres in part by increasing miR-128 level (Fig. 4c).

Based on the above findings, we speculated that BRM270-induced miR-128 overexpression would inhibit A549 cell migration and invasion. We tested this hypothesis with the wound healing and invasion assays. As expected, cells treated with BRM270 and miR-128 mimic showed reduced migration relative to control miRNA-transfected cells, as evidenced by the slower rate of wound closure (Fig. 4d). Additionally, the invasion assay revealed that cells treated with BRM270 and miR-128 had decreased invasive capacity as compared to control cells (Fig. 4e). These data indicate that BRM270 reduces the malignant behavior of A549 cells by inducing miR-128.

### BRM270 suppresses vascular endothelial growth factor (VEGF) signaling in A549 cells

**Fig. 5:**
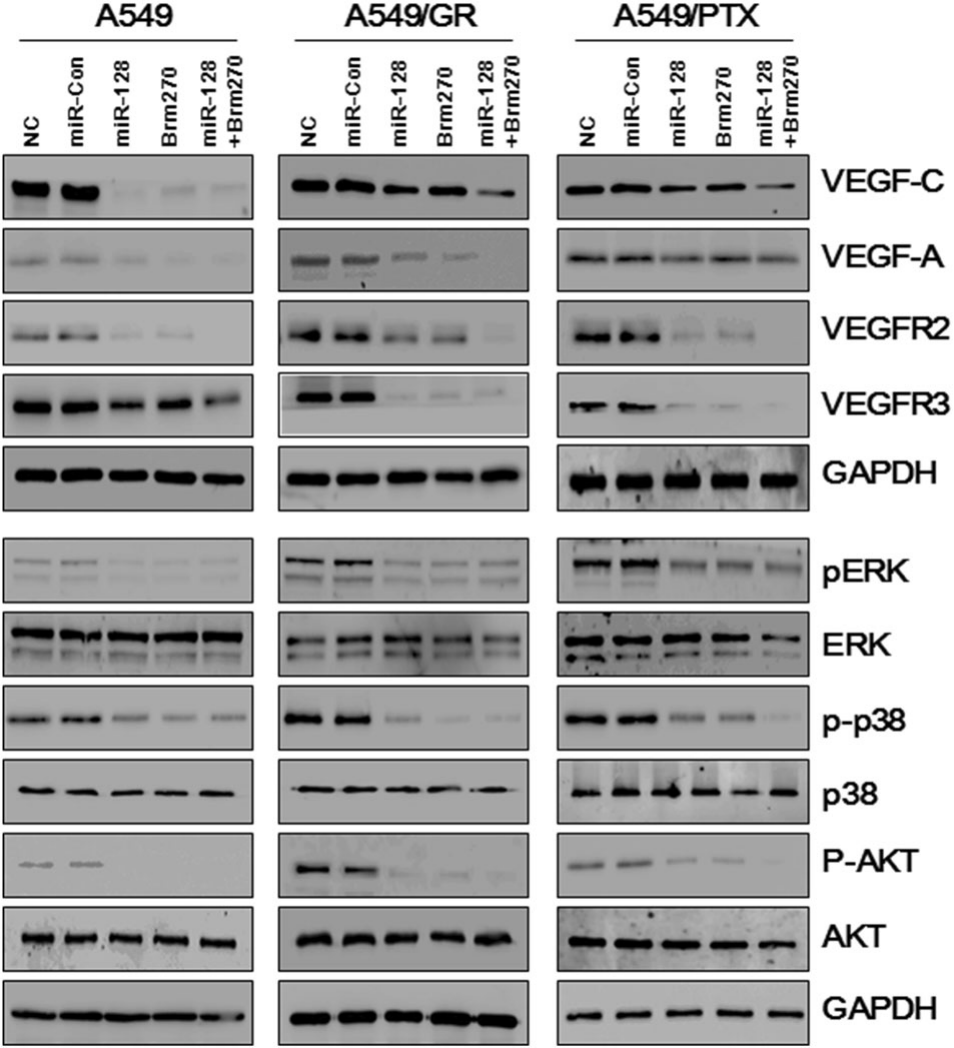
BRM270 directly targets miR-128 and VEGF. Western blot analysis of VEGF-C and VEGF-A, VEGFR2, VEGFR3, and phosphorylated ERK, p38, and AKT levels in A549 cell lines treated with miR-128, BRM270, or both

MiR-128 has been shown to inhibit angiogenesis and tumor growth and block p38/extracellular signal-regulated kinase (ERK)/AKT signaling in cancer cells^9^. Moreover, miR-128 overexpression reversed GR by inhibiting the phosphoinositide 3-kinase (PI3K)/AKT pathway^9^. We therefore examined whether BRM270-induced miR-128 expression affects the levels of VEGF/PI3K/AKT signaling pathway components in A549 cells. BRM270/miR-128 treatment decreased VEGF-C, VEGF-A, VEGF receptor (VEGFR)2 and VEGFR3 expression in all three cell lines (Fig. 5). The combined treatment had a more potent effect than either miR-128 or BRM270 alone. VEGFRs activate the ERK, PI3K/AKT, p38 pathways, which are critical for proliferation of chemoresistant cells. We found here that BRM270/miR-128 treatment decreased ERK p38-mitogen-activated protein kinase and AKT phosphorylation in A549/GR and A549/PTX cells to a greater extent than miR-128 or BRM270 alone. Additionally, the levels of VEGF-C, VEGFR2, phosphorylated ERK, p38, and AKT were similarly expressed in the cells treated with miR-128 inhibitor NC and the cells treated with miR-128 inhibitor. Those expression levels were significantly downregulated by the combined treatment of BRM270 with miR-128 inhibitor compared with the cells treated with miR-128 inhibitor or the cells treated with miR-128 inhibitor NC as observed by western blot (Supplementary Figure 2). Taken as a whole, these observations suggest that BRM270 suppresses VEGFR-induced activation of ERK/p38/AKT signaling via miR-128 activation.

### BRM270 inhibits tumor growth in vivo

**Fig. 6:**
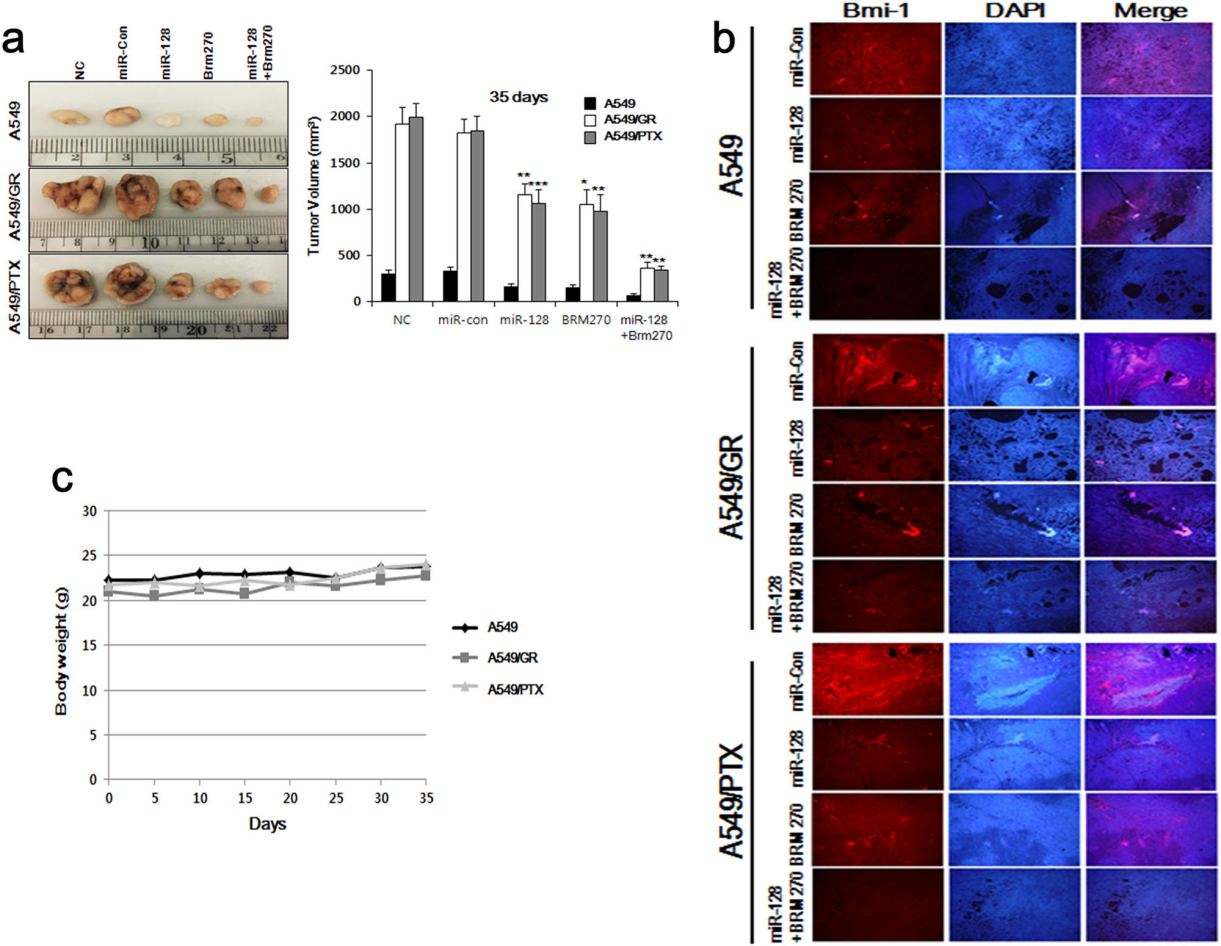
Anti-tumorigenic effects of BRM270 on A549 cell xenografts. **a** Xenografts of A549 cells treated with miR-128, BRM270, or both showed reduced growth relative to those cells treated with either agent alone or left untreated (control). **b** Tumor tissues from xenografts of cells treated with miR-12, BRM270, or both showed reduced Bmi-1 expression by Immunofluorescence. Columns show mean ± SEM. **c** Quantitative analysis of tumor weights. Data represent mean ± SEM (*n* = 5 per group). *P < 0.05, **P < 0.01, ***P < 0.001

We used a xenograft model to evaluate the in vivo effects of BRM270. A549 cell tumors were established in nude mice, which were then divided into the following four groups: negative control, miR-128, BRM270 (1 mg/kg, oral administration via a catheter), or BRM270+miR-128. Mice treated with both miR-128 and BRM270 had smaller tumors than those in other groups (Fig. 6a). We also found Bmi-1 expression was markedly decreased in tumors from mice receiving the combined treatment as compared to other groups, as determined by tissue immunofluorescence (Fig. 6b). Additionally, the effect of miR-128 inhibitor NC, the effect of miR-128 inhibitor, and the combined effect of BRM270+miR-128 inhibitor were observed on A549/GR tumors. Bigger tumors were observed in the mice treated with miR-128 inhibitor and smaller tumors were observed in the mice treated with BRM270+miR-128 inhibitor compared with the mice treated with miR-128 inhibitor NC, as determined by the IRDye^®^ 800CW 2-DG (radiolabeled 2-deoxy-d-glucose (2-DG) based optical imaging of xenograft models with A549/GR tumors (Supplementary Figure 3a, b and c), indicating the inhibition of tumor growth by BRM270 via activation of miR-128. Importantly, BRM270 was not toxic to mice, since there was no weight loss in the treatment group as compared to the negative control mice (Fig. 6c). These results indicate that BRM270 is a safe as well as effective agent for treating chemoresistant lung cancer.

## Discussion

The development of drug resistance is a major obstacle for the effective treatment of human malignancies^18^. Gefitinib and paclitaxel are first-line chemotherapeutic agents used for various cancers. Given their role in the chemoresistance of tumor cells^19^, future research on cancer treatments should focus on miRNAs as targets^7^.

BRM270 is an extract formulated from seven plants (*Saururus chinensis*, *Citrus unshiu Markovich, Aloe vera, Arnebia euchroma, Portulaca oleracea*, *P.vulgaris var. lilacina*, and * Scutellaria baicalensis*) used in traditional Asian medicine that inhibits the proliferation of many types of cancer cell^14^. In the present study, we investigated the mechanism by which BRM270 exerts anti-tumorigenic effects. We found that BRM270 increased miR-128 and inhibited miR-21 expression in A549 cell lines. miR-21 is upregulated in NSCLC and promotes lung cancer cell growth and invasion. We speculate that BRM270 blocks NF-κB signaling, resulting in the inhibition of miR-21 expression. In prostate cancer, the p65 subunit of NF-κB increased miR-21 expression by binding to the miR-21 promoter. On the other hand, miR-21 overexpression decreased gefitinib sensitivity by suppressing phosphatase and tensin homolog, and AKT/ERK pathway activation, whereas miR-21 knockdown restored gefitinib sensitivity via AKT/ERK inhibition.

miR-128 is frequently downregulated in lung cancer^9^; however, it has also been shown to act as a tumor suppressor in several malignancies by inhibiting cell proliferation, migration, and invasion^20^. The reported effects of miR-128 on glioma cells include Bmi-1 downregulation resulting in decreased glioma stem cell self-renewal. miR-128 inhibits cell proliferation by targeting Bmi-1 to suppress neuroblastoma cell motility^21^. Factors that regulate Bmi-1 expression are likely to be critical for normal stem cell maintenance and may thus determine the self-renewal capacity of CSCs^21^. We confirmed that BRM270-induced miR-128 overexpression decreased the protein levels of Bmi-1 and Musashi-1 in A549 cell lines. miR-128 has been shown to abolish chemoresistance by inhibiting PI3K/AKT signaling^19^. Notably, miR-128 was downregulated whereas VEGF-C was upregulated in breast cancer tissue and cells and directly targeted VEGF-C in human bladder cancer cells^22^. VEGFR/VEGF-C modulates tumor invasion and metastasis^23^. High levels of miR-128 inhibited tumor angiogenesis and progression blocked ERK, AKT, and p38 signaling pathways^9^. The results of the present study indicate that miR-128 induced by BRM270 suppresses VEGFR-induced activation of ERK/p38/AKT signaling, as evidenced by the decreases in ERK, p38, and AKT phosphorylation.

In conclusion, BRM270 suppressed proliferation and induced apoptosis in chemoresistant A549 lung adenocarcinoma cells by modulating VEGF/PI3K/AKT signaling via miR-128. We also found that BRM270 had antitumorigenic effects in a mouse xenograft model without apparent toxicity. These findings indicate that BRM270 is a safe and effective alternative to conventional drugs for the treatment of chemoresistant NSCLC.

## Materials and methods

### Cell culture and treatments

A549, A549/GR, and A549/PTX cells were maintained in Roswell Park Memorial Institute 1640 medium (Invitrogen, Carlsbad, CA, USA) containing 10% fetal bovine serum (FBS; Hyclone, Logan, UT, USA), penicillin (100 U/ml), and streptomycin (100 mg/ml). miR-128-overexpressing or inhibition and control miRNA-expressing A549, A549/GR, and A549/PTX cell lines were established by transfection of pCMV-miRNA-128, control pCMV-miRNA (http://www.origene.com/), miRNA-128-inhibitor, and miRNA-control (www.genepharma.com), respectively^9^.

### Flow cytometry

Apoptotic A549, A549/GR, and A549/PTX cells were detected by flow cytometry. Cells were washed with phosphate-buffered saline (PBS) and incubated with Annexin V Binding Buffer (BD Biosciences, Franklin Lakes, NJ, USA), then labeled with Annexin V-fluorescein isothiocyanate (BD Biosciences) as recommended by the manufacturer. Cell cycle analysis was performed as follows: approximately 5 × 10^4^ cells were seeded in six-well culture plates and incubated in complete medium until they reached 70–80% confluence. The cells were harvested, washed twice with ice-cold PBS, and fixed with 70% cold ethanol for 2 h at 4 °C. The different phases of the cell cycle were distinguished by propidium iodide-phycoerythrin staining of nuclei. Fluorescence was detected with a FACS Calibur flow cytometer (BD Biosciences)^24^.

### Sphere formation assay

A549, A549/GR, and A549/PTX cells (2 × 10^3^/well) were seeded in a six-well Ultra Low Cluster plate (Corning Inc., Corning, NY, USA) and cultured in suspension in serum-free Dulbecco’s Modified Eagle’s Medium/F12 (Gibco, Grand Island, NY, USA) containing B27 supplement (1:50; Invitrogen CA, USA), 20 ng/ml EGF (Calbiochem, CA, USA), and 0.5% bovine serum albumin (Sigma-Aldrich, St. Louis, LO, USA) for 10–14 days. The number of cell spheres (defined as spherical, non-adherent cell clusters >100 μm in diameter) was counted and the spheres were imaged with an inverted microscope. Sphere formation efficiency was calculated as colonies/input cells × 100%^4^.

### Xenograft model

Mice were maintained and used for experiments according to a protocol approved by the Institutional Animal Care and Use Committee of Jeju National University (Jeju, Korea). The tumorigenicity of A549, A549/GR, and A549/PTX cells was assayed by subcutaneous inoculation of 1 × 10^5^ cells resuspended in a mixture of 100 µl Matrigel (Sigma-Aldrich, MO, USA) in PBS into the flanks of 8-week-old athymic BALB/c female nude mice (*n* = 5/group). Tumor size was measured at 3-day intervals using calipers (volume = [shortest diameter^2^ × longest diameter]/2) and grafts were removed and photographed 35 days after cell inoculation^24^.

### Data analysis

Statistical analysis was conducted using SPSS v.20.0.1 software (SPSS Inc., Chicago, IL, USA). The *χ*^2^ test or Fisher’s exact test was used as appropriate. *P* < 0.05 was considered statistically significant.

## Electronic supplementary material

Supplementary Information
