# Supplementary material for: BRM270 inhibits cancer stem cell maintenance via microRNA regulation in chemoresistant A549 lung adenocarcinoma cells
Source: Cell Death Dis. 2018 Feb 14;9(2):244. doi: 10.1038/s41419-018-0277-7 (PMC5833813; doi:10.1038/s41419-018-0277-7)
Supplement: Supplementary file 1 — Supplementary Information [file 41419_2018_277_MOESM1_ESM.docx]

**Supplementary Information**

**BRM270 inhibits cancer stem cell maintenance via microRNA regulation in chemoresistant A549 lung adenocarcinoma cells**

**Kwon et.al.**

**Running Title:** BRM270 inhibits CSC in chemoresistant A549 cells

**Supplementary Figure 1 ------------------------------------------------------------------------ 2 page**

**Supplementary Figure 2 ------------------------------------------------------------------------ 3 page**

**Supplementary Figure 3 ------------------------------------------------------------------------ 4 page**

**Supplementary Figure 1**


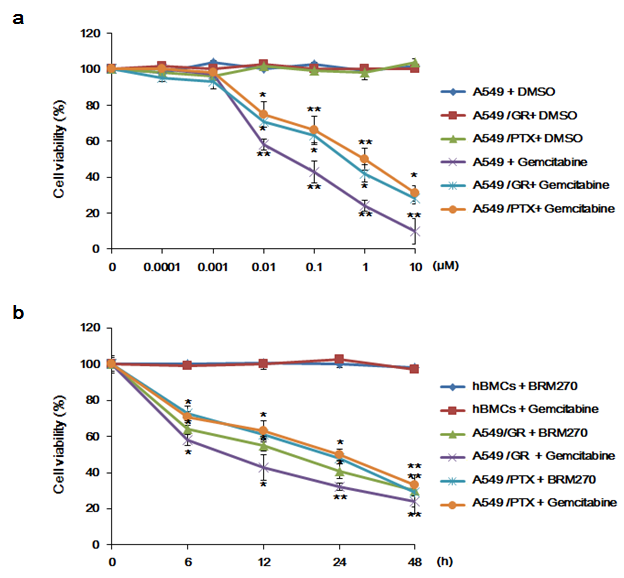


**Supplementary Figure 1** (**a**) Approximately 2 × 10^4^ cells/well were seeded in 96-well plates and treated with Gemcitabine (0, 0.0001, 0.001, 0.01, 0.1, 1, 10 µM) for 24h; 0.1% dimethyl sulfoxide in medium was used as a control. (**b**) Time course studies of BRM270 (120µg) with compared to Gemcitabine (0.1µM) against both A549/GR and A549/PTX cells up to 48 h. **P < 0.05, **P < 0.01.*

**Supplementary Figure 2**


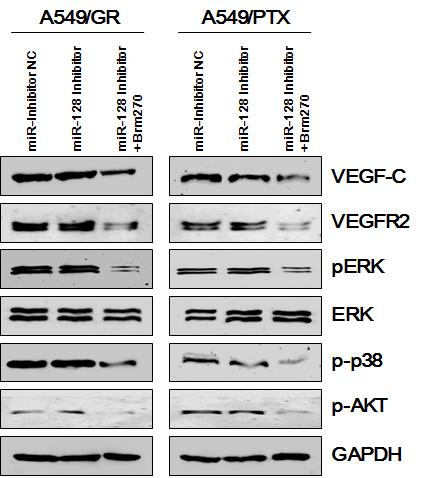


**Supplementary Figure 2** Western blot analysis of VEGF-C, VEGFR2, and phosphorylated ERK, p38, and AKT levels in A549/GR and A549/PTX cells treated with miR-128 inhibitor NC, miR-128 inhibitor and miR-128 inhibitor+BRM270.

**Supplementary Figure 3**


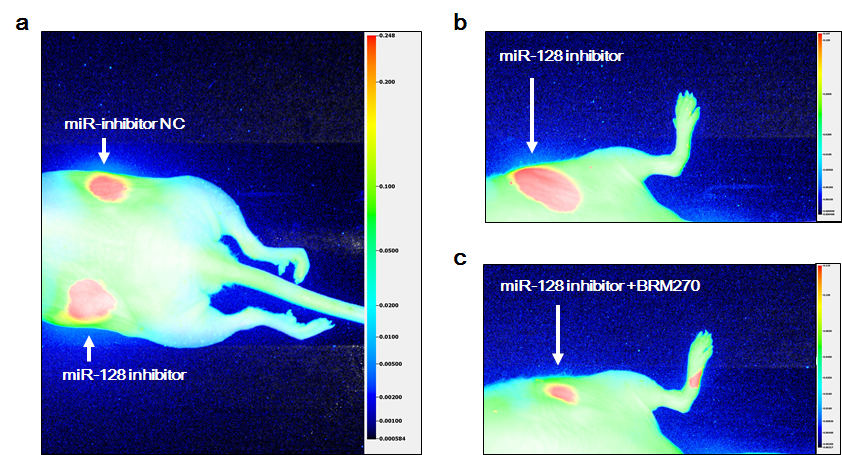


**Supplementary Figure 3** BRM270 halts tumor formation in IRDye® 800CW 2-DG optical probe guided molecular imaging xenograft model. (**a**) Radiolabeled 2-deoxy-D-glucose (2-DG) based optical imaging of the xenograft models with A549/GR tumors treated with miR-128 inhibitor NC and miR-128 inhibitor (**b**) with miR-128 inhibitor (**c**) with miR-128 inhibitor+BRM270.
